# Diagnostic Accuracy of Pleural Effusion Mononuclear Cells/Leukocyte Ratio in Tuberculous Pleurisy

**DOI:** 10.3389/fmed.2021.639061

**Published:** 2021-03-18

**Authors:** Xiaoli Lei, Junli Wang, Zhigang Yang

**Affiliations:** ^1^Department of Pulmonary and Critical Care Medicine, Henan Provincial People's Hospital, Zhengzhou, China; ^2^Department of Cardiopulmonary Function, Fuwai Central China Cardiovascular Hospital, Zhengzhou, China

**Keywords:** tuberculous pleurisy, mononuclear cell/leukocyte ratio, TB pleural effusion, adenosine deaminase, diagnostic study

## Abstract

**Background:** Tuberculous pleurisy (TBP) is a common clinical type of tuberculosis (TB) in China. TBP is difficult to diagnose. Whether the mononuclear cell/leukocyte (MNC/LEU) ratio in pleural effusion can contribute to accurate TBP diagnosis remains yet unclear.

**Objective:** To explore the diagnostic value of MNC/LEU ratio in pleural effusion for TBP in China.

**Methods:** This study was a retrospective case-control study involving 406 patients with pleural effusion of unknown cause who were hospitalized at the Henan Provincial People's Hospital. Using histopathological examination of thoracoscopic pleural biopsy as the gold standard for TBP diagnosis, a final total of 215 subjects were included in this study including 91 cases of TBP and 124 cases of non-TBP. The receiver operating characteristic (ROC) curve of pleural effusion MNC/LEU ratio for TBP diagnosis was plotted and the area under curve (AUC) and the optimal cutoff value were determined. In addition, the sensitivity, specificity and accuracy of the MNC/LEU ratio at the optimal cutoff value for TBP diagnosis were also evaluated.

**Results:** The MNC/LEU ratio was significantly higher in TB pleural effusion [95.9% (89.7–98.0%)] than in non-TB pleural effusion [77.8% (39.3–93.2%)] (*P* < 0.001). The AUC was 0.776 (95% CI, 0.714–0.830), and the sensitivity, specificity and accuracy for TBP diagnosis at the 93.7% cutoff value were 64.83%, 79.03%, and 0.730, respectively.

**Conclusion:** The pleural effusion MNC/LEU ratio may be a new and valuable laboratory indicator for the diagnosis of tuberculous pleurisy in China.

## Introduction

Tuberculosis (TB) is one of the top ten causes of death worldwide and has been the leading cause of death due to a single infectious disease since 2007 (1). Tuberculous pleurisy (TBP) is the second most common extrapulmonary TB following lymph node TB (2) accounting for 26% of extrapulmonary TB in China (3). Although TBP is common in clinical practice, its diagnosis remains difficult for clinicians.

Microbiology and pleural histopathology are the accepted gold standards for TBP diagnosis (4, 5). However, the low abundance of Mycobacterium tuberculosis (MTB) in the pleural effusion and pleural tissue often result in low MTB culture positive rate and long culture cycle. In fact, the MTB positive rates of pleural effusion and cultured pleural tissue are only 40 and 65%, respectively (6). As a result, clinical diagnosis of TBP mainly relies on histopathological examination of pleural tissues obtained by biopsy. However, the MTB positive rate of histopathological examination by percutaneous acupuncture pleural biopsy was only 57.5% (7), and the blindness was large and the sampling was limited, and complications such as pleural reaction and pneumothorax were prone to occur. On the other hand, video-assisted thoracoscopy allows accurate multi-site biopsies of suspicious lesions under direct vision and provides a nearly 100% diagnostic rate of TBP (8). Video-assisted thoracoscopy has been widely used in tertiary hospitals of China. However, most TBP patients in China seek treatment in primary and secondary hospitals, which do not perform thoracoscopy due to the lack of trained thoracoscopic physicians and valuable thoracoscopic equipment. Although the quantitative MTB/rifampicin resistance nucleic acid amplification test (Xpert MTB/RIF) has emerged as a promising molecular biological detection technique in recent years, the test is expensive, and has a low sensitivity of 37–51.4% (9, 10) and has limited screening for TBP (11). Therefore, more accurate, safer, less invasive and rapid diagnostic methods are urgently needed for TBP.

TBP is characterized by MTB infection and lymphocyte and mononuclear cell infiltration in the pleural membrane, which consequently result in exudation, hyperplasia, and necrotizing inflammation. MTB infection in the pleural membrane is strongly hinted when the lymphocyte/leukocyte (LYM/LEU) ratio is greater than 75% (12). In addition, studies have reported that the combination of monocyte/leukocyte (MONO/LEU) ratio and pleural effusion Adenosine deaminase (ADA) test had up to 98% specificity in TBP diagnosis (13, 14). However, monocytes and lymphocytes in pleural effusions are usually not differentiated by the automated hematology analyzer currently used in the clinical setting, and the default mode of detection only includes leukocyte count, multinuclear cell count, mononuclear cell count, multinuclear cell/leukocyte ratio and mononuclear cell/leukocyte ratio. Mononuclear cells in the body fluid are mainly comprised of lymphocytes and monocytes. Our previous work has confirmed that pleural effusion mononuclear cell count had 76% sensitivity, 57% specificity and 66% accuracy in TBP diagnosis at the optimal cutoff value of 969.6 × 10^6^ cells/L (15). Whether pleural effusion mononuclear cell/leukocyte (MNC/LEU) ratio has more diagnostic value for TBP is unclear. Therefore, this study aimed to evaluate the diagnostic value of pleural effusion MNC/LEU ratio for TBP and to provide a more accurate, simple, rapid and minimally invasive diagnostic method for TBP.

## Research Objects and Methods

### Subjects

The clinical data of 406 patients with pleural effusion of unknown cause who were hospitalized at the Department of Pulmonary and Critical Care Medicine of Henan Provincial People's Hospital between January 1, 2014 and September 1, 2019 were reviewed.

#### Inclusion Criteria

(1) Presence of unilateral or bilateral pleural effusion confirmed by chest x-ray, chest CT or ultrasound; (2) Histopathological examination of pleural biopsy obtained by thoracoscopy; (3) Known MNC/LEU ratio in pleural effusion; (4) Determined etiology of pleural effusion; (5) Complete clinical data.

#### Exclusion Criteria

(1) Absence of pleural biopsy by thoracoscopy or pleural histopathological results; (2) Undetermined etiology of pleural effusion; (3) Incomplete clinical data.

#### Diagnostic Criteria for TBP

Diagnostic criteria for TBP (16, 17): (1) Caseous necrotic granuloma, PCR-TB-DNA (+) and/or acid-fast staining (+) by pleural histopathology; (2) Detection of acid-fast bacilli in pleural tissue or pleural effusion specimens and identification of MTB by culture.

#### Diagnostic Criteria for Non-TB Pleural Effusion

Lack of microbiological or histological evidence of TBP. All cases of neoplastic pleural effusion were confirmed by histopathology or pathological cytology. Other causes of pleural effusion were definitively diagnosed according to the patients' symptoms, signs, microbiology and imaging results, autoantibody profile and clinical follow-up data.

#### Grouping

Patients were divided into the TBP group and non-TBP group based on their pleural histopathology results.

### Methods

#### Data Collection

A clinical data collection form was developed and data were collected through the Digital Hospital Information Management System of Henan Provincial People's Hospital. If a patient's data in the system was incomplete, the original medical record was consulted in the Medical Record Room of the hospital and the missing data were filled. Data that were collected from the electronic medical records of patients include: (1) Basic information of patients including gender, age and underlying disease; (2) Histopathological results of thoracoscopic pleural biopsy; (3) Pleural effusion ADA; (4) MNC/LEU ratio of pleural effusion.

#### Thoracoscopy

Thoracoscopic procedures are detailed in our previous publication (15).

#### Determination of MNC/LEU Ratio in Pleural Effusion

About 8–10 ml of pleural effusion was collected from the patients and the MNC/LEU ratio was determined by a laboratory physician using the Sysmex XN-9000 automated hematology analyzer (Sysmex Corporation, Japan) according to the instrument instructions.

#### Calculation of the Sample Size

Sample size (*n*) was calculated according to the formula shown below where u_α_ was 1.96, δ was 0.1, and p was the sensitivity or specificity (18). The minimum calculated sample size for this study was 106 patients, including 37 in the case group and 69 in the control group. A final total of 215 patients were included in this study.

$$\begin{array}{l} n=\left[u\alpha^{2}p\left(1-p\right)\right]/\delta^{2} \end{array}$$

#### Blinding

All data including MNC/LEU ratio and ADA were determined and entered into the computer independently by two laboratory technicians who were unaware of the pleural biopsy results. All pleural histopathological results were judged by two independent pathologists and any discrepancy was resolved by a third pathologist. The pathologists were all unaware of the MNC/LEU ratio.

#### Ethics

The protocol of this retrospective case-control study was submitted and approved by the Ethics Committee of Henan People's Hospital prior to the start of the study. This study was approved to not require the signing of the informed consent.

#### Statistical Analysis

Measured data that conform to the normal distribution were expressed as mean ± standard deviation (X ± S), and those that do not conform to the normal distribution were expressed as median (interquartile range, IQR). Measured data were compared using two independent sample t test or Mann-Whitney U test. Count data were expressed as frequency or percentage and compared using the chi-square test or Fisher's exact test. The diagnostic power of pleural effusion MNC/LEU ratio or ADA was plotted as a receiver operating characteristic (ROC) curve to determine the optimal cutoff value for the test parameters and evaluation indicators related to diagnostic accuracy studies such as sensitivity, specificity and accuracy. *P* < 0.05 was considered statistically significant. All the statistical analyses were performed using MedCalc and SPSS (version 23.0).

**Figure 1:**
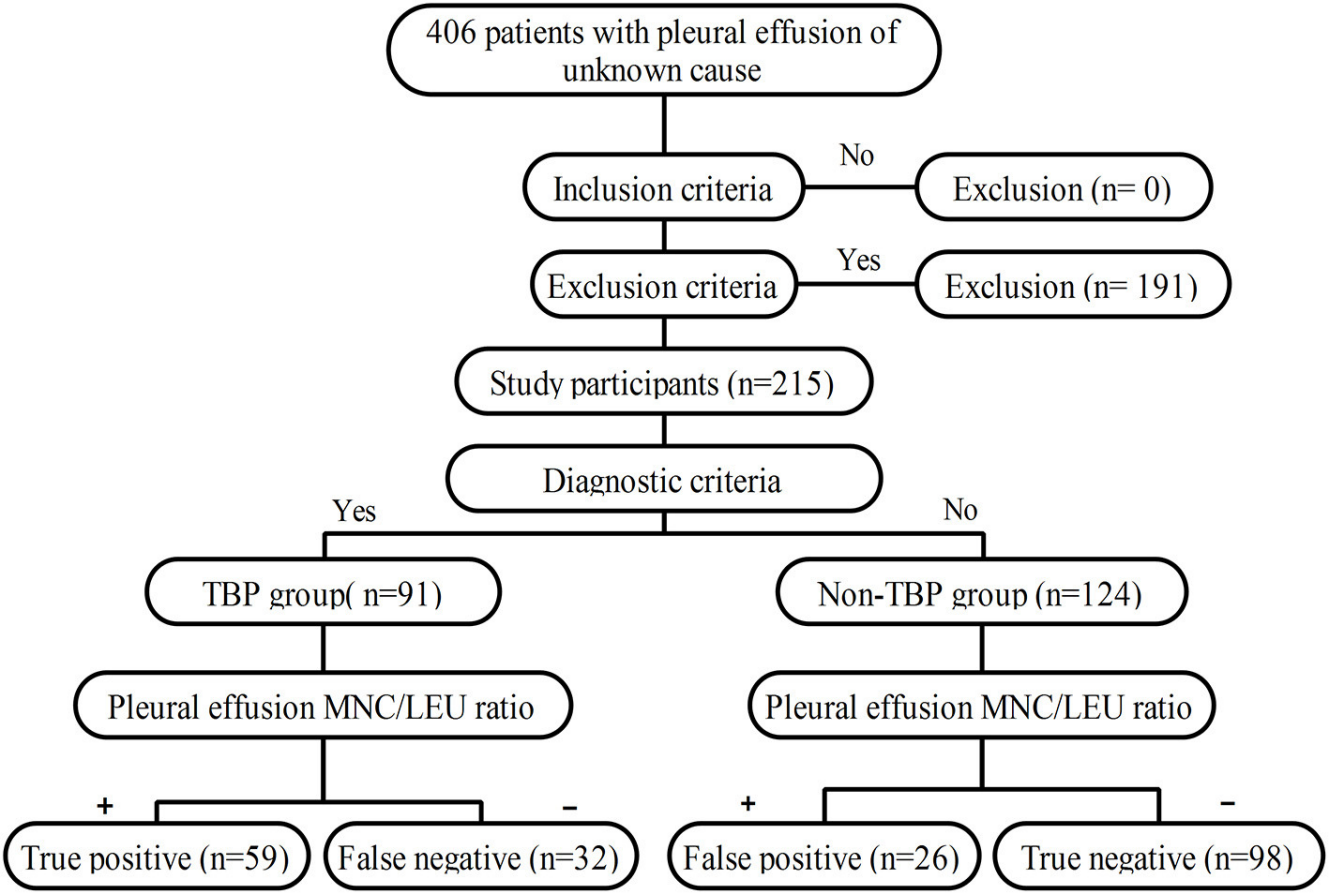
Flowchart of the study.

The study workflow is shown in Figure 1.

## Results

### Clinical Characteristics, Underlying Diseases, and Etiological Classification of Subjects

The clinical data of 406 patients with pleural effusion of unknown cause were reviewed. Of these patients, 191 were excluded according to the exclusion criteria and 215 were included according to the inclusion criteria. According to the histopathological results of pleural biopsy, 91 of the 215 patients were confirmed with TBP and the other 124 had non-TBP. The TBP group consisted of 67 males and 24 females with a median age of 51.00 (26.00–62.00) years. The non-TBP group consisted of 84 males and 40 females with a median age of 62.50 (52.00–70.00) years. The median age of TBP patients was significantly lower than that of non-TBP patients (*Z* = 5.256, *P* < 0.001). There was no significant difference in gender between the two groups (*X*^2^ = 0.869, *P* = 0.351). However, the incidences of underlying diseases such as diabetes and coronary heart disease were significantly higher in the non-TBP group than in the TBP group (*X*^2^ = 5.112, *P* = 0.024 and *X*^2^ = 9.216, *P* = 0.002, respectively). In the TBP group, there were 5 cases with concomitant pulmonary TB. In the non-TB group, there were 69 cases (55.6%) with malignant pleural effusion, 15 cases (12.1%) with empyema, 28 cases (22.6%) with parapneumonic effusion, and 12 cases (9.7%) with pleural effusion of other causes (Tables 1, 2).

**Table 1:** Clinical characteristics and underlying diseases of 215 subjects.

**Characteristics**	**TBP group** **(*n* = 91)**	**Non-TBP group** **(*n* = 124)**	***P*-value**
Age (median, interquartile range)	51.00 (26.00, 62.00)	62.50 (52.00, 70.00)	0.000
**Gender (*****n*****, %)**			
Male	67 (73.6)	84 (67.7)	0.351
Female	24 (26.4)	40 (32.3)	
**Underlying disease (*****n*****, %)**			
Alcohol consumption	25 (27.5)	41 (33.1)	0.380
Smoking	36 (39.6)	59 (47.6)	0.242
Diabetes	5(5.5)	19 (15.3)	0.024
Hypertension	24 (26.4)	36 (29.0)	0.668
Arrhythmia	2 (2.2)	10 (8.1)	0.064
Coronary heart disease	1 (1.1)	15 (12.1)	0.002
Chronic gastritis	1 (1.1)	7 (5.6)	0.169
Brain infarction	6 (6.6)	11 (8.9)	0.541
COPD	1 (1.1)	6 (4.8)	0.255
Bronchial asthma	1 (1.1)	2 (1.6)	1.000
Rheumatic disease	1 (1.1)	7 (5.6)	0.169
Hyperthyroidism	0 (0)	2 (1.6)	0.509
Malignant tumor	3 (3.3)	3 (2.4)	1.000
Chronic hepatitis B	5 (5.5)	4 (3.2)	0.634
Previous TB infection history	0 (0)	6 (4.8)	0.087
HIV infection	0 (0)	0 (0)	1.000
Prior TB treatment	8 (8.8)	14 (11.3)	0.550
Prior hormonal theapy	1 (1.1)	2 (1.6)	1.000

*TBP, Tuberculous pleurisy; Non-TBP, Non-tuberculous pleurisy; COPD, Chronic obstructive pulmonary disease; HIV, Human immunodeficiency virus*.

**Table 2:** Etiological classification and composition of 215 subjects.

**Disease classification**	**Number**	**Proportion**
TBP	91	
TBP with pulmonary TB	5	5.5%(5/91)
Non-TBP	124	
Malignant pleural effusion	69	55.6%(69/124)
Parapneumonic effusions	28	22.6%(28/124)
Empyema	15	12.1%(15/124)
Chylothorax	1	0.8%(1/124)
Pulmonary embolism	1	0.8%(1/124)
Pulmonary contusion	1	0.8%(1/124)
Liver cirrhosis	2	1.6%(2/124)
Microscopic polyangiitis	1	0.8%(1/124)
Acute glomerulonephritis	1	0.8%(1/124)
Constrictive pericarditis	1	0.8%(1/124)
Cardiac insufficiency	3	2.4%(3/124)
Hypoproteinemia	1	0.8%(1/124)

*TBP, Tuberculous pleurisy; Non-TBP, Non-tuberculous pleurisy*.

### Comparison of Pleural Effusion MNC/LEU Ratio Between TBP Group and Non-TBP Group

**Figure 2:**
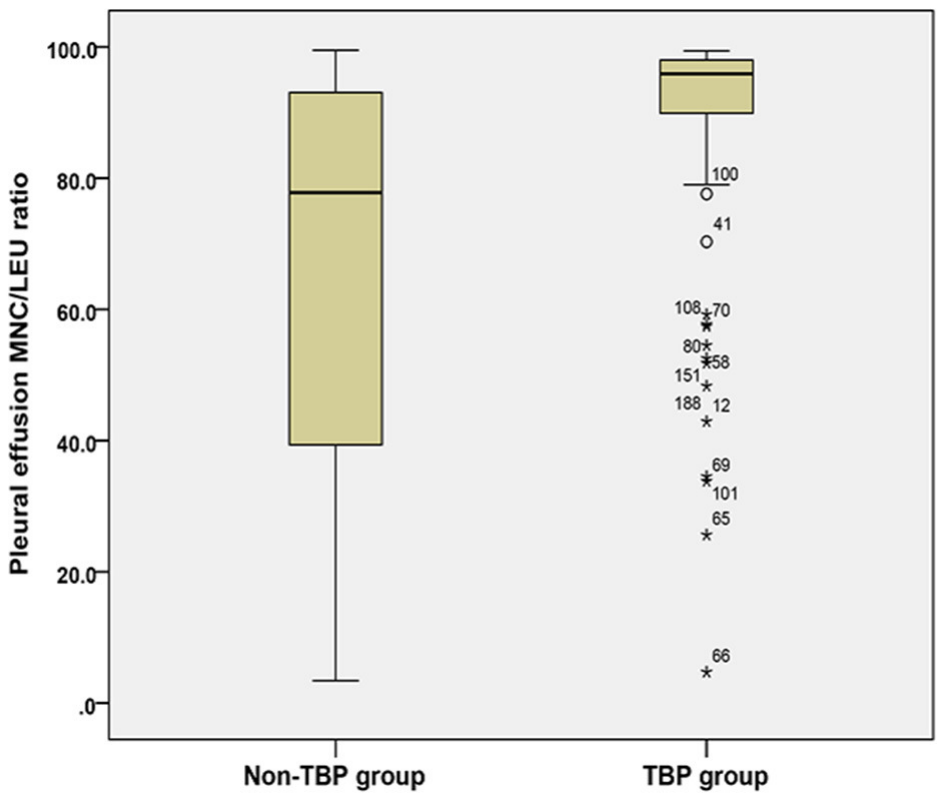
Comparison of pleural effusion MNC/LEU ratio between the TBP group and non-TBP group. Figure shows the median and inter quartile range (IQR) of the ratio of mononuclear cells to leukocytes in pleural effusion between the tuberculous pleurisy group and the non-tuberculous pleurisy group. MNC/LEU, mononuclear cell/leukocyte; TBP, Tuberculous pleurisy; Non-TBP, Non-tuberculous pleurisy. *means outlier.

The pleural effusion MNC/LEU ratio was significantly higher in the TB group [95.9% (89.7–98.0%)] than in the non-TB group [77.8% (39.3–93.2%)] (*Z* = 6.902, *P* < 0.001) (Table 3; Figure 2).

**Table 3:** Comparison of pleural effusion MNC/LEU ratio between the TBP group and non-TBP group.

**Group**	**Number of cases**	**MNC/LEU ratio (median, interquartile range), (%)**	***Z* value**	***P*-value**
TBP group	91	95.9 (89.7, 98.0)	6.902	0.000
Non-TBP group	124	77.8 (39.3, 93.2)		

*MNC/LEU, mononuclear cell/leukocyte; TBP, Tuberculous pleurisy; Non-TBP, Non-tuberculous pleurisy*.

### Diagnostic Value of Pleural Effusion ADA for TBP

The optimal cutoff value for ADA was >26 U/L when the maximum value of Youden index was 0.607. The sensitivity (Sen.), specificity (Spe.), positive predictive value (+PV), negative predictive value (–PV), positive likelihood ratio (+LR), negative likelihood ratio (–LR) and accuracy of pleural effusion ADA in TBP diagnosis at the 26U/L cutoff value were 85.71%, 75.00%, 71.6%, 87.7%, 3.43, 0.19, and 0.795, respectively (Table 4).

**Table 4:** Comparison of diagnostic power of pleural effusion MNC/LEU ratio or ADA for TBP.

	**MNC/LEU**	**95% CI**	**ADA**	**95% CI**
Cutoff value	>93.7%	–	>26U/L	–
Sen. (%)	64.8	54.1–74.6	85.71	76.8–92.2
Sep. (%)	79.03	70.8–85.8	75.00	66.4–82.3
+PV(%))	69.4	58.5–79.0	71.6	62.1–79.8
–PV(%)	75.4	67.1–82.5	87.7	79.9–93.3
+LR	3.09	2.1–4.5	3.43	2.5–4.7
–LR	0.44	0.3–0.6	0.19	0.1–0.3
Accuracy	0.73	–	0.795	–
AUC	0.776	0.712–0.839	0.810	0.749-0.870

*MNC/LEU, mononuclear cell/leukocyte; ADA, adenosine deaminase; TBP, Tuberculous pleurisy; Sen, sensitivity; Spe, specificity; +PV, positive predictive value; –PV, negative predictive value; +LR, positive likelihood ratio; –LR, negative likelihood ratio; AUC, area under curve*.

### Diagnostic Value of Pleural Effusion MNC/LEU Ratio for TBP

The optimal cutoff value for MNC/LEU ratio was 93.7% when the maximum value of Youden index was 0.439. The Sen., Spe., +PV, –PV, +LR, –LR, and accuracy of MNC/LEU ratio in TBP diagnosis at the 93.7% cutoff value were 64.8%, 79.03%, 69.4%, 75.4%, 3.09, 0.44, and 0.73, respectively (Table 4).

### Comparison of the Area Under Curve Between Pleural Effusion ADA and MNC/LEU Ratio for TBP Diagnosis

**Figure 3:**
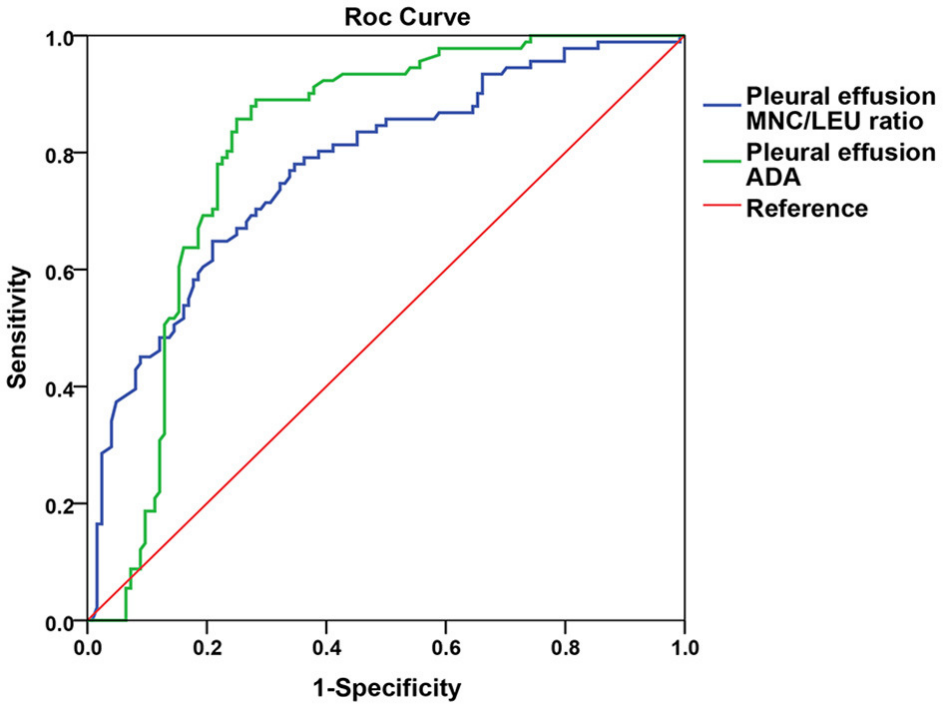
Comparison of the AUC between pleural effusion ADA and MNC/LEU ratio for TBP diagnosis. Figure shows that the AUC of pleural effusion ADA and MNC/LEU ratio were 0.8 10 (0.749–0.870), 0.776 (0.712–0.839), respectively, and there was no statistical difference between the two groups. *P* = 0.446. AUC, area under curve; MNC/LEU, mononuclear cell/leukocyte; ADA, adenosine deaminase; TBP, Tuberculous pleurisy.

The AUC of pleural effusion ADA and MNC/LEU ratio were 0.810 (0.749–0.870) and 0.776 (0.712–0.839), respectively, and there was no statistical difference between the two groups (*P* = 0.446) (Figure 3).

## Discussion

In this study, we found that the pleural effusion MNC/LEU ratio was significantly higher in the TBP group than in the non-TBP group [95.9% (89.7–98.0%) vs. 77.8% (39.3–93.2%), *p* < 0.001], which suggests that MNC/LEU ratio can be used as a valuable laboratory marker for the diagnosis of TBP. The result of our study is similar to those of previous studies by He et al. (14) and Zhang and Tong (13). He et al. (14) found that the TB pleural effusion group had significantly higher monocyte count and median of MONO/LEU ratio (86.10%) than the tumor group (35.80%). Similarly, Zhang and Tong (13) reported that when pleural effusion MONO/LEU ratio was 84.5%, its sensitivity and specificity for the differential diagnosis of TB and non-TB pleural effusion were 63.3 and 53.7%, respectively. Those results mentioned above suggest that MNC/LEU ratio may be valuable in the diagnosis of tuberculous pleurisy. We looked further and found that the AUC of pleural effusion MNC/LEU ratio for TBP diagnosis was 0.776 (95% CI, 0.714–0.830), which suggested that MNC/LEU ratio had diagnostic value in TBP. When the maximum Youden index was 0.439, the optimal diagnostic cutoff value for MNC/LEU ratio was 93.7%. The median of sensitivity, specificity, positive predictive value, negative predictive value, positive likelihood ratio, negative predictive ratio and accuracy at this cutoff value were 64.8%, 79.0%, 69.4%, 75.4%, 3.09, 0.44, and 0.73, respectively. Furthermore, we found that greater than 93.7% of MNC/LEU ratio can be used as a new diagnostic marker for TBP with an accuracy of 73% in patients with unknown pleural effusion. This is better than the absolute mononuclear cell count proposed in our previous work, which has a diagnostic accuracy of only 66% (15).

The number of lymphocytes and monocytes was reported to be significantly increased in TBP effusion (11, 12, 19) and the LYM/LEU ratio and MONO/LEU ratio have demonstrated certain diagnostic value in TBP. A previous study showed that a pleural fluid LYM/LEU ratio greater than 64% had 89.1% sensitivity and 76.4% specificity in TBP diagnosis (19). Furthermore, the diagnostic specificity of MONO/LEU ratio was significantly improved (as high as 98%) when used in combination with the pleural effusion ADA test. These findings suggested that both LYM/LEU ratio and MONO/LEU ratio are useful markers for TBP diagnosis. It is important to note that lymphocytes and monocytes are usually enumerated together as mononuclear cells by the automated hematology analyzer during routine clinical testing. This method is simple, rapid and feasible (20, 21), and has been used in routine clinical pleural effusion tests. However, the diagnostic value of pleural effusion MNC/LEU ratio for TBP has not been reported in the literature. That's why we did this study for the first time. Our study demonstrated for the first time that MNC/LEU ratio can be used to diagnose tuberculous pleurisy.

We also compared the diagnostic value of MNC/LEU ratio and ADA in pleural effusion for tuberculous pleurisy, the latter being known to be a valuable marker for the diagnosis of tuberculous pleurisy. Our study found that the AUC of pleural effusion ADA and MNC/LEU ratio were 0.810 (0.749–0.870) and 0.776 (0.712–0.839), respectively, and there was no statistical difference between the two groups (*P* = 0.446). Since the determination of pleural effusion MNC/LEU ratio is simpler, faster, cheaper than that of ADA, it is worthy of further clinical study and validation.

Our study showed that the median age of patients was lower in the TBP group than in the non-TBP group. In line with our finding, a study by Chakrabarti and Davies (22) showed that TBP was more prevalent in adolescents and young adults than in the elderly. In addition, we found that the non-TBP group had higher incidences of chronic diseases (diabetes mellitus and coronary heart disease) at baseline than the TBP group. But, Mamaev et al. (23) reported that the incidence of pleurisy was lower in TB patients without diabetes than in those with diabetes, suggesting that diabetes had an effect on the development of TBP. On the other hand, the effect of coronary heart disease on TBP is currently unclear and will therefore need to be further investigated.

There were several limitations in this study. First, this was a retrospective case-control study rather than a concurrent cross-sectional study, which is the best type of diagnostic study. As a result, the conclusion of this study may have less power than that of a cross-sectional study. Second, subjects in this study were inpatients from tertiary hospitals in Henan Province of China and had relatively severe conditions. Since this was a single-center study, there may be selection bias in the subjects examined. Therefore, the diagnostic accuracy of pleural effusion MNC/LEU ratio for TBP will need to be further validated in subsequent multicenter concurrent cross-sectional studies.

To sum up, the pleural effusion MNC/LEU ratio may be a new and valuable laboratory indicator for the diagnosis of tuberculous pleurisy in China. Because MNC/LEU ratio determination of pleural effusion is simple, accurate and practical, it is worthy of further verification and promotion.

## Data Availability Statement

The raw data supporting the conclusions of this article will be made available by the authors, without undue reservation.

## Ethics Statement

The studies involving human participants were reviewed and approved by The Ethics Committee of Henan Provincial People's Hospital. Written informed consent for participation was not required for this study in accordance with the national legislation and the institutional requirements.

## Author Contributions

ZY contributed conception and design of the study. JW and XL collected the data, organized the database, and analyzed the data. XL, JW, and ZY wrote the first draft of the manuscript. All authors contributed to the final version of the manuscript.

## Conflict of Interest

The authors declare that the research was conducted in the absence of any commercial or financial relationships that could be construed as a potential conflict of interest.
